# Supplementary material for: Are carbon emissions trading and green financial instruments synergistic? -Comprehensive quantitative research based on content analysis
Source: PLoS One. 2024 Mar 7;19(3):e0298601. doi: 10.1371/journal.pone.0298601 (PMC10919601; doi:10.1371/journal.pone.0298601)
Supplement: S1 Data — (ZIP) [file pone.0298601.s001.zip › Data/地级市Z_F工作报告.zip!/░▓╗╒/╕╖╤⌠╩╨/└┤╫╘╣1⁄2╓┌║┼í╛┬φ┐╦╩2╛▌═°í┐_2009.docx]

各位代表：

现在，我代表市人民政府，向大会作政府工作报告，请予审议，并请市政协委员和其他列席人员提出意见。

一、2008年工作回顾

刚刚过去的2008年，是我市改革发展进程中非常重要而又极不平凡的一年。在中共阜阳市委的正确领导下，全市人民认真贯彻落实党的十七大、十七届三中全会和胡锦涛总书记、温家宝总理视察阜阳重要讲话精神，深入学习实践科学发展观，积极应对冰雪灾害、手足口病等突发事件和严峻挑战，攻坚克难，拼搏奋进，较好完成了市三届人大三次会议确定的主要目标任务。

经济平稳较快发展。初步预计，全市生产总值540亿元，比上年增长12％，连续5年保持两位数增长。实现财政收入50.8亿元，增长26.3％。农村经济全面发展，全年农业总产值310亿元，增长8％。粮食生产连续5年获得丰收，总产在全省率先突破百亿斤大关。畜牧养殖业发展势头良好，规模养殖比重达55％，比上年提高10个百分点。工业经济持续较快增长，规模以上工业实现增加值123亿元，增长24％。工业经济综合效益指数达到252％，高于全省平均水平30个百分点。产值超1亿元企业新增7家，超10亿元的新增4家。第三产业实现增加值195亿元，增长12％。社会消费品零售总额237亿元，增长23％。城镇居民人均可支配收入11727元，农民人均纯收入3100元，分别增长8％和17％。

改革开放不断深入。农村综合改革稳步推进，土地流转制度改革有序进行，集体林权制度主体改革基本完成，畜牧兽医体制改革顺利实施。国有企业改革力度加大，307户企业完成改制任务，156户企业进入改制程序。义务教育经费保障机制改革全面推行，新型农村合作医疗实现全覆盖。行政审批制度改革继续深化，行政服务中心功能逐步完善。金融改革步伐加快，农业银行、农村信用社改革扎实推进，邮政储蓄银行小额信贷等业务不断拓展。对外贸易稳步增长，实现进出口总额1.85亿美元，增长17％。招商引资成效明显，引进市外项目917个，到位资金112亿元、增长21％，实际利用外资8500万美元、增长30％。

项目建设成效明显。全年固定资产投资完成190亿元，增长20％。“6611”工程完成投资120亿元，占年度计划的108％，其中“861”行动计划项目完成投资56亿元，占年度计划的125％。重点项目建设进展顺利，合阜高速公路建成通车，沙颍河初步治理工程基本完成，颍上船闸顺利通航；福润公司年屠宰300万头生猪生产线、临化公司航天炉建成试生产；口孜东煤矿、京九铁路电气化、昊源合成氨下游产品系列开发，以及沙颍河耿楼枢纽、行流大桥、颍上二桥、太和三桥等项目建设进度加快；阜六高速公路恢复建设，漯阜铁路复线电气化、阜阳机场提升通航保障能力改造工程及濛洼中岗大桥开工建设；阜淮铁路电气化改造、阜阳船闸、沙颍河航道整治、阜新高速、阜六铁路等项目前期工作进展顺利。

城乡建设步伐加快。《阜阳城市总体规划（2007—2020年）》获得省政府批准，阜阳皖西北中心城市的地位进一步确立。阜城基础设施建设力度加大，南京路泉河大桥建成通车，西二环路、颍上路等6条城市主干道改造完成，北三环路及颍河大桥开工建设，阜亳高速公路连接线建设步伐加快，商厦路口地下人防主体工程完工，河滨路工程建设基本完成，颍河大桥、泉河二桥加固维修工程竣工通车。三角洲移民迁建工程基本完成，三角洲公园开工建设，泉河风景带建设进度加快，西清河景观工程、窦棚沟清淤疏浚工程和黑龙潭泵站建设完成。市体育中心运动场工程基本完工，阜阳城市生活垃圾卫生填埋场开工建设，新建和改造城市公厕202座。“五城同创”活动扎实开展，城市管理力度进一步加大，城市面貌明显改观，荣获“全省创建文明城市工作先进市”称号。县城和小城镇建设稳步推进，城镇化水平进一步提高。污染减排工作力度加大，在淮河流域率先实现县县有污水处理厂的目标。新农村建设“百村示范、千村整治”行动计划扎实推进，中心村、居民点建设规划逐步实施。

社会事业全面进步。创新型阜阳建设稳步实施，产学研结合步伐加快，科技创新能力逐步提高，涌现出一批创新型企业和科研成果。信息化建设力度加大，国家级农村信息化服务试点工作深入推进，荣获“数字安徽”建设先进单位称号。阜城中小学布点规划加快实施，义务教育均衡发展方案全面启动，农村中小学D类危房改造任务基本完成。职业教育基础能力得到加强，高中、成人、民办和高等教育稳步发展。竞技体育水平进一步提高，阜阳籍运动员邓琳琳荣获北京奥运会女子体操金牌，实现了我市奥运会奖牌和金牌“零”的突破。广播电视“村村通”和文化惠民工程深入实施，我市与国家有关单位联合拍摄的电影《农民工》被列为纪念改革开放30周年优秀重点献礼影片。旅游经济健康发展，颍州西湖风景名胜区规划工作扎实推进。计划生育奖励扶助政策得到落实,人口和计划生育工作水平逐步提高。第一次全国污染源普查工作有序开展。妇女儿童、老年人及残疾人权益得到保障。国防后备力量和武警、消防、人防工作得到加强。新闻出版、海关、出入境检验检疫、物价、外事、侨务、民族、宗教、对台、地震、气象、审计、统计、文物保护、档案、地方志、地名等工作也都取得了新的成绩。

一年来，主要做了以下五个方面工作：

（一）坚持第一要务，大力促进经济平稳较快发展。面对复杂多变的宏观经济形势，始终坚持把发展作为阜阳最大的政治、最硬的道理和最根本的任务，采取切实有效措施，化解突出矛盾，着力在抢抓机遇中加快发展。针对上半年物价过快上涨、企业生产成本上升等问题，切实加强经济运行调节和煤电油运调度，加大价格监管力度，扎实推进建设用地置换和节能降耗等项工作，成功举办了银企对接会，缓解了建设用地、环境容量和资金紧张的矛盾，保持了经济平稳较快增长的良好势头。进入下半年以来，面对金融危机的不利影响，认真贯彻宏观调控政策，制定落实促进经济平稳较快增长的实施意见。针对企业生产经营出现的困难，坚持分类指导、一企一策，实行市级领导联系重点企业制度，对重点企业进行对口帮扶，扩大了中小企业担保基金规模，建立了贷款风险补偿金，取消了部分行政事业性收费，清理和规范了经营性、服务性收费。制定了增强自主创新能力的实施细则，大力推进技术创新。同时，抢抓中央扩内需、保增长的政策机遇，高度重视项目谋划，成立专门班子，扎实做好项目编报争取工作，累计编报项目318个，第一批有37个项目得到国家批准，争取投资3.5亿元，为经济持续发展打下了良好基础。

（二）坚持扩大开放，强力推进招商引资工作。始终把招商引资作为加快发展的重要抓手，抢抓沿海发达地区产业转移机遇，不断创新招商方式，强化工作调度，完善激励机制，招商引资力度进一步加大。坚持市级领导带头招商、部门负责人外出招商、小分队驻点招商，扎实开展招商引资月活动，组织参加了徽商大会、中部博览会、厦门贸洽会和东莞投资环境说明会、温州瑞安招商会等重大招商经贸活动，香港南益集团、森仕集团等一批知名企业投资项目落户我市。注重发挥园区招商引资重要载体作用，先后4次召开工业园区现场会，加大园区基础设施建设力度，努力增强园区的吸引力和承载力。成功举办了第二届皖西北(阜阳)投资贸易洽谈会，签约合同类项目21个、投资总额39亿元。

（三）坚持以人为本，全力维护社会和谐稳定。面对各种困难和严峻挑战，我们始终坚持人民至上、生命至上，建立健全应急管理机制，着力提高应急能力，有效处置了各类突发公共事件。去年初冰雪灾害发生后，我们果断应对，紧急行动，千方百计保畅通、保供应、保民生、保稳定，最大限度地减少了损失。面对突如其来的手足口病疫情，迅速建立高效运转的组织指挥体系，全面落实包保责任制，扎实开展爱国卫生运动，全力以赴抓防控，不惜代价救患儿，有效控制了疫情，受到省委、省政府和国家卫生部的充分肯定。汶川地震发生后，全市上下紧急动员，迅速行动，广泛开展捐赠活动，累计捐赠款物8000多万元。同时，派出医疗救援小分队和青年志愿者奔赴灾区，开展伤员救治和对口帮扶。妥善处置三鹿婴幼儿奶粉事件，全面开展问题奶粉清查，及时免费筛查救治患儿。高度重视生产安全、食品安全和药品安全，深入开展肉类食品市场及保健品安全专项整治。扎实推进平安阜阳建设，认真做好维稳和奥运安保工作，广泛开展领导干部开门接访、带案下访活动，加大矛盾纠纷排查调处力度，切实抓好社会治安综合治理和禁毒工作，维护了社会稳定大局。

（四）坚持民生优先，着力解决事关群众利益的突出问题。认真按照胡锦涛总书记一项一项抓好落实、不断抓出成效的要求，深入组织实施18项民生工程，全年共投入20.6亿元，惠及800多万城乡居民。对农村饮水安全等工程类项目，严格执行项目标准，坚持一月一调度，强化督促检查，有力推动了项目的顺利实施。高度重视社会保障和就业再就业工作，加强社会保险扩面征缴，全市城镇职工基本养老、基本医疗、失业、生育保险和城镇居民医疗保险人数均超额完成省下达目标任务。低保提标扩面任务全面完成，城乡救助体系逐步健全，被征地农民基本养老保险启动实施。在全省率先将未参保集体企业职工纳入基本养老保险范畴，妥善解决了集体企业退休人员的养老问题。高度关注弱势群体，重大传染病医疗救治、生活救助政策得到有效落实。认真做好返乡农民工工作，及时制定措施，进行调查登记，开展免费培训，促进了返乡农民工的就业和创业。

（五）坚持依法行政，努力加强政府自身建设。围绕建设责任政府、服务政府和法治政府，认真贯彻《国务院关于加强市县政府依法行政的决定》，推进管理创新，狠抓责任落实，努力提高服务发展、服务基层、服务群众的能力和水平。全面实施《政府信息公开条例》，政务信息公开范围不断扩大。坚持重大事项集体决策制度，扎实推进政府决策的科学化、民主化。加强基层政权建设，顺利完成第七届村民委员会换届选举工作。各级政府自觉接受人大法律监督、政协民主监督，认真办理人大代表议案、建议和政协委员提案，广泛听取各民主党派、工商联、无党派人士及各人民团体的建议和意见，鼓励支持人民群众、新闻媒体对政府工作进行监督。大力优化发展环境，深入推进机关效能建设，组织市直执法窗口单位赴合肥市行政服务中心开展对口学习，行政审批效率得到提高。扎实开展反腐倡廉制度建设推进年活动，加大廉政建设力度，强化经济责任审计和行政监察，政府工作人员廉政意识进一步增强，从政行为进一步规范。

在肯定成绩的同时，我们也清醒地认识到，我市经济社会发展中还存在一些突出矛盾和问题。一是发展不快、结构不优的问题依然突出。二产比重偏低，规模以上企业较少，民营经济的支柱地位还需进一步加强。节能减排压力加大，人口、资源、环境矛盾突出。加之受宏观经济形势的影响，加快发展的压力增大。二是县域经济发展不平衡，农村基础设施薄弱，抵御自然灾害的能力不强，农业持续增长和农民增收的难度加大。三是随着增值税转型等税制改革的实施，财政减收因素增多，刚性支出加大，收支矛盾进一步加剧。四是城镇化水平偏低，城市功能不强，文明创建任务艰巨。五是社会事业发展相对滞后，就业和社会保障任务繁重，部分城乡居民的生活还比较困难，社会热点难点问题和不稳定因素仍然较多。六是发展环境有待进一步优化，有些职能部门不作为、慢作为、乱作为问题严重，形式主义、官僚主义和铺张浪费等现象还不同程度存在。同时，政府工作也存在着不少差距和不足，职能转变还不能适应市场经济发展的需要，领导科学发展和驾驭复杂局面的能力亟待进一步提高。对此，我们一定高度重视，认真加以解决。

各位代表，过去的一年，是难忘的一年，是拼搏的一年，也是有着丰硕成果的一年。在面临年初的冰雪灾害、上半年的手足口病、下半年的金融危机等带来的诸多不利因素的情况下，我们能够取得这样的成绩，确实来之不易。这是省委、省政府和市委正确领导的结果，是市几套班子大力支持的结果，也是全市广大干部群众齐心协力、共克时艰的结果。在此，我代表市人民政府，向辛勤工作在全市各条战线上的广大干部群众，向给予政府工作大力支持与监督的人大代表、政协委员、民主党派、工商联、无党派人士、各人民团体、新闻媒体和其他各界人士，向关心支持阜阳建设的离退休老干部、老职工，向为阜阳改革发展稳定作出积极贡献的解放军指战员、武警官兵、预备役部队、政法干警，向中央和省驻阜单位及所有关心支持阜阳建设与发展的同志们、朋友们，向在我市创业的境内外投资者、建设者，致以崇高的敬意和衷心的感谢！

二、2009年工作安排

2009年是国际国内经济发展面临严峻挑战的一年，也是我市推进“十一五”规划顺利实施的关键一年。当前，国际金融危机还在演变，实体经济受到的冲击可能进一步加剧，国际市场需求仍在下降，全国、全省经济增长速度放缓，对我市经济的冲击也逐步显现。但全国经济发展的基本趋势没有改变，全省加快发展的总体态势没有改变。经过连续几年的努力，我市经济抗风险的能力也明显增强。随着一批重点基础设施项目的开工建设，一批工业项目的投产达产，我市经济持续快速发展的基础进一步巩固。尤其是去年下半年以来，中央和省一系列扩内需、保增长政策措施的相继出台，省委、省政府支持皖北地区发展意见的全面落实，都给我市加快发展带来了难得机遇。总体上，信心与压力同在，机遇和挑战并存。政府工作的指导思想是：全面贯彻党的十七大、十七届三中全会和中央、全省经济工作会议精神，以开展深入学习实践科学发展观活动为动力，围绕推进科学发展、和谐发展、跨越发展，全力以赴扩大内需，坚定不移推进经济结构调整和发展方式转变，大力实施工业突破和商贸物流提升行动，努力壮大现代农业和县域经济，着力提升“6611”工程和招商引资水平，切实加强资源节约和环境保护，更加重视改善民生和促进社会和谐，努力推动经济社会又好又快发展。

综合考虑各种因素，主要预期目标是：全市生产总值增长11%；财政收入增长12％以上；固定资产投资增长25％以上；社会消费品零售总额增长15％以上；招商引资总额增长20％以上；进出口总额增长15％；城镇居民人均可支配收入增长8％；农民人均纯收入增长6％以上；人口自然增长率控制在8.5‰以内；城镇登记失业率控制在4％以内；单位生产总值能耗降低7％左右；主要污染物排放总量减少2％。

围绕上述目标，重点抓好以下八个方面的工作：

（一）以优化升级为重点，推动工业经济实现新突破。切实把发展工业摆在更加突出的位置，深入实施工业突破四年行动计划，强力推进工业结构优化升级，进一步提升工业对经济增长的主动力。一是加大优势产业和重点企业扶持力度。加快实施颍河工业经济增长带、首太工业走廊发展规划，加大对煤电、化工、食品、有色金属、木材加工产业扶持力度，着力培育医药、塑料、纺织、服装产业，鼓励支持机械、电子产业加快发展。继续实行市级领导联系重点企业制度，坚持一企一策、一品一策，综合运用财税、金融、价格等调控手段，帮助企业解决资金周转、生产经营、项目建设等方面的难题，引导受金融危机影响较小的食品、医药、烟酒等企业生产更多适销对路产品。切实加大对40户重点骨干企业的支持力度，促使谢桥煤矿、刘庄煤矿扩大生产，确保口孜东煤矿建成投产，加快推进罗园煤矿建设，实施华源纺织集团等退城进园、退二进三项目，扩大昊源集团、临化公司与山西晋煤集团以及开乐公司与中国航空工业集团的战略合作，力争全年新增销售收入超10亿元企业2户、超20亿元和超30亿元企业2户。同时，进一步落实扶持措施，加快中小企业发展。用足用好外贸促进政策，努力保持出口稳定增长。二是加大企业创新力度。鼓励企业利用市场竞争的倒逼机制，积极引进先进技术和设备，加快技术改造和产业升级步伐。引导企业推进管理创新，苦练内功，降低成本，提高经济效益和竞争力。加快重点技改项目建设，力争昊源集团年产20万吨合成氨原料路线改造及年产30万吨尿素扩建工程、临化公司航天炉二期工程、阜南沪千公司年产22万立方米中高密度纤维板、太和贝克药业新药生产等一批重点技改项目及早竣工投产。三是加大工业园区建设力度。坚持把工业园区作为实施工业突破的重要平台，继续实行两个月一汇报、半年一检查，全力推动园区建设加快发展。利用省支持园区建设专项资金，加快完善园区基础设施，提升优势产业聚集能力。重点支持阜阳经济技术开发区加快建设步伐，积极发展高新技术和优势产业，不断壮大实力，努力建设成为承接产业转移的示范区、特色产业的聚集区。依托资源优势打造一批特色县域工业园区，促进区域特色工业经济快速发展。进一步抓好阜阳循环经济园、太和城关镇工业园、界首光武塑料工业园和阜南黄岗柳编工业园等工业集中区建设。四是加大国有企业改革力度。以建立现代企业制度为目标，以盘活资产、增强活力为核心，积极引进战略投资者，加强市属国有企业改革成本筹措支付平台建设，多渠道筹集改革资金，加快156户企业改制进度，启动32户企业改制工作，年内基本完成国有企业改革目标。五是加大节能减排工作力度。严格落实目标责任制，实施节能十项攻坚，加强27户重点耗能企业和重点用能单位的日常监察，确保完成年度目标任务。加大城市污水管网建设力度，提高污水收集率和处理能力。扎实开展泉北污水处理厂前期工作，开工建设颍东污水处理厂，力争年内建成运行。加快阜阳城市生活垃圾卫生填埋场建设，确保尽快投入使用。推进垃圾发电项目和中水再生利用项目前期工作。

（二）以农民增收为核心，推动“三农”工作开创新局面。认真落实党在农村的各项方针政策，进一步深化农村改革，加快发展现代农业，扎实推进社会主义新农村建设，千方百计增加农民收入。一是大力发展种植业促增收。加快规划建设淮河粮食功能主产区，着力打造“江淮粮仓”品牌。继续抓好10个优势农产品基地建设，重点抓好“2358”示范工程。大力实施小麦高产攻关活动、水稻产业提升行动和玉米振兴计划，重点抓好275万亩小麦、25万亩水稻和140万亩玉米核心示范区建设。加快推进国家级农产品检测中心建设。新建农产品标准化生产基地50万亩以上，争创省级、国家级标准化基地2个以上，认证无公害农产品、绿色食品30个以上。大力调整种植业结构，培育特色农业，发展一村一品、一乡一业。进一步抓好“菜篮子”工程。二是突出养殖业促增收。认真落实财政以奖代补等扶持畜牧业发展的各项政策，大力推进标准化规模养殖和健康养殖。实施牛羊产业可持续发展、生猪产业标准化、家禽产业化和渔业富民工程，突出抓好规模化养殖、重大动物疫病防控、无规定动物疫病区建设和畜禽改良体系建设，发展现代畜牧业，不断提高规模化养殖比重，壮大养殖规模。三是推进农业产业化促增收。开展龙头企业推进年活动，继续实施农业产业化财政以奖代补政策，重点支持市级以上龙头企业做大做强。鼓励农户与龙头企业、中介组织形成不同形式的利益联合体，让农民更多地分享农产品加工、销售环节的利润。支持农村综合信息服务站和农民专业合作社建设。同时，依托丰富的农副产品优势，广泛开展农业招商活动，吸引更多的全国知名龙头企业来阜投资。四是壮大劳务经济促增收。认真落实促进农民工就业的各项政策措施，深入实施“阳光工程”，大力开展农民工技能培训，着力提高农民工就业能力。组织开展全市企业用工与返乡农民工岗位对接活动，促进返乡农民工就业。加强与劳务输入地区的联系和衔接，引导农民工有序外出务工，促使外出务工人员由体力型向技能型转变，由短期输出型向稳定就业型转变，着力打造劳务品牌，努力增加农民劳务收入。实施“凤还巢”工程，建设农民工返乡创业园，鼓励支持农民工返乡创业。五是深化农村综合改革促增收。积极探索建立农村基层工作新机制。按照依法自愿有偿原则，鼓励农民以转包、转让、出租、互换、股份合作等多种形式流转土地承包经营权，探索建立农村土地承包经营权流转市场。加强农村公路、农田水利、电网、饮水、通讯等基础设施建设。加快农田林网和绿色长廊工程建设，努力提高森林覆盖率。加大农村宅基地管理力度，切实把新农村建设与土地复垦整理、旧村庄改造有机结合起来，进一步做好土地置换工作，扩大城乡建设用地增减挂钩试点，努力改善村容村貌。

（三）以商贸物流中心建设为依托，推动第三产业焕发新活力。围绕建设辐射几千万人口的现代商贸物流中心，启动商贸物流提升行动，大力实施商贸流通百亿元工程，切实提高服务业对经济增长的贡献率。一是加快发展核心商业区和物流园区。重点扶持人民路传统商业区，开工建设香港财富广场、千百意商城二期、阜阳商厦四期扩建项目，着力构建阜城商业核心区。实施红星美凯龙、大润发等项目建设，打造阜城西二环商业区。完善提高三十里铺物流加工园区和辛桥物流园区功能，积极扶持华联、商厦物流中心建设，加快雨润食品产业园、中储粮农产品物流园、阜阳粮食产业园等大型物流项目建设。二是切实加强专业市场建设。进一步提升瑶海大市场、汇鑫家居城、皖西北商贸城等专业市场的档次，启动阜阳农产品批发市场扩建项目，重点培育阜阳临沂综合商城、开发区汽贸汽配等专业市场。加快推进县域特色专业市场建设，发展壮大太和人发、界首再生塑料、阜南柳编、颍上大米、临泉瓦店山羊和脱水蔬菜等县域特色市场。三是繁荣农村消费市场。进一步健全农村流通网络，新建和改造一批农家店和农村商品配送中心，增加配送品种，增强综合服务功能。继续实施“万村千乡”市场工程和“新网工程”，组织开展家电下乡、农机下乡等活动，进一步拉动农村消费。四是千方百计扩大消费。积极落实消费政策，提振消费信心，培育消费热点，扩大消费需求。进一步加快服务业发展，积极发展面向投资需求的生产性服务业和面向消费需求的生活性服务业，发展文化、教育培训和社区服务等需求潜力大的产业。完善城市社区便民服务设施，促进城市耐用消费品升级换代。认真落实稳定和刺激房地产业发展的相关措施，增加保障性住房供给，加快老城区和城中村改造步伐，促进房地产市场稳步健康发展。大力发展旅游经济，加快提升风景名胜区档次，高起点规划建设颍州西湖等主要旅游景区，进一步推进旅游目的地建设，着力培育阜阳生态文化旅游产业。

（四）以项目建设为支撑，推动投资规模迈上新台阶。抢抓国家扩大内需的政策机遇，千方百计加大投资力度，确保固定资产投资有一个较快增长。一是加强项目谋划争取投资。继续围绕民生、公益、农业、节能、技改和重大基础设施建设，抓紧谋划、储备一批项目，做实做细项目前期工作，简化项目申报程序，全力做好项目争取工作，主动搞好项目和资金对接，努力争取更多的项目投资。二是突出项目建设落实投资。今年初步安排“6611”工程项目377个，年度计划投资160亿元，其中列入省“861”行动计划项目92个，年度计划投资78亿元。进一步完善项目工作推进机制，继续实行市级领导联系重大项目制度，切实强化项目调度，确保一批重点项目建设取得实质性进展。开工建设阜淮铁路电气化、阜六铁路、阜新高速和阜阳港等重大项目，实施阜阳电网扩建工程，加快漯阜铁路复线电气化和阜阳机场提升通航保障能力改造工程进度，建成濛洼中岗大桥，确保阜六高速按时通车。切实加强项目管理，强化资金监管，确保工程质量。三是加大招商力度引进投资。充分发挥阜阳区位、交通、资源和市场优势，紧紧抓住沿海地区产业资本转移的机遇，重点围绕煤电、化工、医药、木材和食品加工等产业，加大招商引资组织领导和工作落实力度。积极参与泛长三角分工与合作，努力在承接沿海地区产业转移上取得新进展。进一步改进招商方式，破解招商难题，继续坚持市级领导带头招商、部门负责人外出招商、小分队驻点招商，推进全民招商，加大产业招商、园区招商、企业招商、会展招商和以商招商力度。认真组织参加徽商大会等重大招商活动，办好第三届皖西北（阜阳）投资贸易洽谈会。四是激活社会资本扩大投资。扎实开展全民创业行动，大力发展非公有制经济。进一步降低门槛，扩大向民间资本开放的领域和行业，引导民间资本投向政府鼓励项目和符合国家产业政策的领域，参与民生工程、基础设施和生态环境建设，努力把民间资金优势转变为发展优势。

（五）以中心城市建设为目标，推动城乡面貌发生新变化。认真组织实施阜阳城市总体规划，进一步加大投入、扩张规模、完善功能、提升品位，加快中心城市建设步伐。一是高起点规划，统领城市发展。继续完善城市规划体系，完成阜阳市土地利用总体规划修编和专项规划、控制性详规编制。按照“以河西区为中心，颍河、泉河为纽带，三区四片组团式”的城市空间布局结构，合理安排城市空间拓展时序。加快县城、中心城镇、建制镇规划编制。坚持依法管理，加强规划监督，切实保障规划的权威性和连续性。二是高规格建设，完善城市功能。进一步加大路网建设力度，拉大城市框架。实施南京路南延和三清路网、七渔河路网建设，建成河滨路西段和阜亳高速公路连接线，打通颍上北路、双清路，加快北三环路及颍河大桥工程进度，改造阜王路等城市主干道，加强出口路改造和整治力度。完善城区供水管网，增强供水功能，及早实现市自来水公司和二水厂并网。加快天然气管网和加气站建设，提高城市供气能力。进一步加大小街巷和城市公厕改造力度。三是高标准美化，改善城市环境。围绕建设滨水园林城市，加强城市园林绿化，改善城市生态环境。加大沟河塘综合治理力度，推进一道河、西城河、七里长沟和中清河、西清河二期综合治理工程，完成华桥沟泵站建设，争取扩容七里长沟泵站，改善内河水质，增强城市防洪排涝能力。加快泉河风景带建设进度，扩建南城河景观带，高标准建设三角洲公园和西城河景观带，规划设计颍河风景带，着力打造城区特色景观。进一步抓好单位、小区和道路绿化，提高绿化覆盖率和人均公共绿地面积。继续实施城市亮化工程。四是高水平管理，塑造城市形象。扎实推进“五城同创”，全面落实创建工作实施方案，加大创建省级文明城市力度，抓好市民素质提升、市容综合整治、基层文明创建和违法建设拆除等项工作，建立健全城市管理长效机制，提高城市管理水平，提升城市品位。进一步加快4县城关镇、界首城区和小城镇建设步伐，加速城镇化进程，逐步形成布局合理、功能明确、结构完善的城镇体系。继续实施“百村示范、千村整治”行动计划，重点抓好省级新农村示范乡镇建设。

（六）以财政金融为抓手，推动经济保障能力得到新提升。认真落实积极的财政政策和适度宽松的货币政策，切实提高运用财政金融工具的能力，努力破解发展中的资金难题，实现财政金融与经济的良性互动。一是强化公共财政职能。依法加强税收征管，健全协税护税体系，挖掘增收潜力，努力应收尽收。进一步规范非税收入管理，推进非税收入征缴信息化改革，促进财政收入较快增长。严格支出管理，压缩一般性支出，集中有限财力保改善民生，保法定支出，保经济增长。发挥财政资金杠杆作用，拉动金融机构扩大对中小企业的信贷投放。进一步加强和完善筹融资平台建设，做好企业债券发行工作，为经济社会发展提供资金保障。认真实施增值税转型改革和成品油税费改革，深化部门预算、“收支两条线”管理和县乡财政管理体制改革，完善政府采购、国库集中支付制度。二是增强金融对经济工作的支持功能。扎实推进金融创新，引导金融机构扩大信贷投放，改善和强化政银企合作，力争贷款规模有较大增长。积极引进境内外金融机构设立分支机构，支持徽商银行加快县域网点布局。加快农村信用社向农村合作银行和农村商业银行过渡，开展村镇银行、小额贷款公司、农村互助基金组织试点，扩大政策性农业保险试点范围，建立农民信贷风险补偿基金和农户小额贷款贴息资金，推进农村房屋权属登记发证和抵押贷款工作。高度重视金融生态环境建设，认真开展金融生态环境县和信用村镇创建活动，切实防范金融风险，维护金融稳定。

（七）以民生工程为载体，推动和谐阜阳建设再创新成绩。更加注重改善民生，着力打造平安阜阳，促进社会和谐。一是巩固提升民生工程。按照省政府统一部署，组织实施28项民生工程。进一步加大投入，整合资源，建立稳定的多元筹资机制，及时足额落实配套资金，重点抓好工程类项目的建设。加强协调调度，强化督促检查，健全科学的绩效考核评价管理制度，确保民生工程扎实推进。二是高度重视就业和再就业工作。切实把扩大就业摆在更加突出的位置，实施更加积极的就业政策，千方百计增加就业岗位。坚持以创业带动就业，强化职业技能培训和就业援助，落实零就业家庭等困难群众的就业帮扶政策，健全覆盖城乡的公共就业服务体系。统筹做好高校毕业生、转业退伍军人、被征地农民等各类人员就业工作。加大劳动监察力度，引导企业履行社会责任，鼓励支持企业吸纳、安置更多的劳动者，力争全年新增城镇就业岗位5万个、下岗失业人员再就业1.3万人。完善劳动关系协调机制，维护劳动者合法权益。三是加快城乡社会保障体系建设。加强社会保险扩面工作，有序推进非公有制经济组织、城镇灵活就业人员和农民工参加社会保险。扩大城镇职工基本养老保险覆盖面，逐步做实养老保险个人帐户，提高企业退休人员基本养老金标准。全面建立城镇居民基本医疗保险制度，巩固完善新型农村合作医疗制度，加快实施被征地农民养老保险制度，探索城乡社会养老保险制度有效衔接的办法。扎实推进社会救助体系建设，提高对低收入家庭等困难群体的补贴和救助标准。进一步加大扶贫开发力度，着力解决农村困难人口的生活问题。四是切实提高社会管理水平。建立健全突发事件应对机制，完善各级各类应急预案，切实增强处置能力。严格落实安全生产责任制，深入开展安全生产专项整治，加大隐患排查和治理力度，强化安全监管，坚决遏制重特大事故的发生。集中开展违法使用食品添加剂专项整治行动，加大食品、药品、保健品监管力度，建立健全长效机制。加强和改进信访工作，完善社会矛盾纠纷排查调处机制，继续开展领导干部开门接访、带案下访，着力解决群众关注的热点难点问题和信访突出问题。深入开展平安阜阳创建活动，加强社会治安综合治理，加大扫黑除恶、打击毒品犯罪和“两抢一盗”力度，切实维护社会和谐稳定。

（八）以维护群众利益为根本，推动社会事业取得新进展。坚持协调发展、全面进步，高度重视和认真解决群众最关心、最直接、最现实的利益问题，让人民群众共享改革发展成果。一是优先发展教育事业。健全城乡义务教育免费政策和经费保障机制，落实义务教育学校实施绩效工资制度。深入实施阜城中小学布点规划，加快推进中小学校标准化建设，全面推行素质教育，加强薄弱学校建设，推动义务教育均衡发展。突出发展职业教育，打造阜阳职教品牌。大力普及高中和学前教育，加强特殊教育工作，规范和促进民办教育，加快推进阜阳大学、阜阳技师学院、阜阳师范专科学校和阜阳职教园区筹建工作。全面建立各教育阶段家庭经济困难学生资助体系，认真做好进城务工人员子女和留守儿童教育工作。二是加快建设创新型阜阳。认真落实推进自主创新的各项政策措施，加大科技投入，推进产学研结合，发展高新技术产业，提高自主创新能力。牢固树立人才是第一资源的理念，切实加大人才强市战略实施力度。加强知识产权保护，大力培养和引进高层次创新人才。深入推进信息化建设，加快普及电子政务，实施无线宽带城市建设，进一步做好农村信息化服务试点工作，建立完善全市名特优产品电子商务平台。三是大力繁荣文化体育事业。推进文化体制改革，挖掘和保护文化资源，发展文化产业，繁荣文艺创作。广泛开展群众性文化活动，深入实施“文化惠民”工程，加快推进阜阳文化艺术中心和乡镇综合文化站、“农家书屋”建设，扎实开展网吧、娱乐场所、电子游戏室等各类文化市场的专项治理和“扫黄打非”行动。开展文物普查，重视文物保护，制定实施城隍庙等保护性修复方案。积极备战省十二届运动会，广泛开展全民健身运动，推进农民体育健身工程，加快市体育中心建设，提升竞技体育水平。深入开展精神文明创建活动，加强社会公德、职业道德、家庭美德教育和青少年思想道德建设，扎实推进诚信阜阳建设。四是完善公共卫生服务体系。推进城镇卫生医疗体制改革，加强城市社区卫生服务体系建设，健全农村三级医疗卫生服务网络，提高医疗卫生水平。深入开展爱国卫生运动，加强对艾滋病等重大传染疾病的防治和救助工作，完善重大疾病防控体系和应急机制。五是扎实推进生态阜阳建设。大力发展循环经济，培育壮大一批循环经济型企业和园区。加大国土资源和环境保护执法监管力度，严格环境准入，促进产业结构调整。加强农村环境保护工作，切实解决危害群众健康和影响可持续发展的突出环境问题，进一步改善和提升环境质量，努力实现节约发展、清洁发展、可持续发展。六是加强人口和计划生育工作。建立健全依法管理、村民自治、优质服务、政策推动、综合治理的人口和计划生育工作新机制，突出抓好人口大县、后进乡村和流动人口的计划生育工作，扩大计划生育长效节育措施奖励扶持范围，千方百计提高出生人口政策符合率、长效节育措施落实率和统计数据准确率，努力降低出生人口性别比，不断提高人口素质。同时，大力推进基层民主政治建设，加大城市社区建设工作力度，积极开展农村社区建设试点。继续做好第二次全国经济普查和调查统计工作。扎实开展法律服务“进千村入万户”活动。进一步做好新形势下的民族宗教工作。继续深化殡葬制度改革。大力发展残疾人、红十字和福利慈善事业，保障妇女和未成年人权益。加强国防动员、民兵预备役和人防工作，支持驻阜部队建设，积极开展双拥共建活动，巩固发展军政军民团结。充分发挥老干部在“三个文明”建设中的作用，支持关工委、老龄委和工会、共青团、妇联工作，继续做好广播电视、新闻出版、海关、出入境检验检疫、外事、对台、侨务、审计、档案、物价、地名、地方志、地震、气象等项工作。

各位代表，展望2009年，发展的压力很大，面临的挑战严峻。要达到预期目标，完成全年任务，我们既要有忧患意识，更要有机遇意识；既要沉着应对，更要迅速行动。全市上下只要团结一致、凝心聚力，敢于拼搏、勇于奋进，因势利导、脚踏实地，就一定能够变压力为动力、化挑战为机遇，把宏观环境带来的不利影响降到最低程度，把经济平稳较快发展的良好势头保持在最佳状态。对此，我们充满信心。

三、切实加强政府自身建设

新的形势、新的挑战对政府工作提出了新的更高的要求。必须切实加强政府自身建设，不断提升科学行政、民主行政、依法行政的能力和水平。

（一）坚持科学理政，进一步提高科学发展的能力。各级政府及其工作人员要深入学习实践科学发展观，准确把握科学发展观的深刻内涵、精神实质和根本要求，以科学发展观武装头脑、指导实践、推动工作。要认真学习现代金融、现代科技、国际经济、法律法规和现代管理知识，努力形成适应形势任务发展变化的知识结构，切实提高驾驭市场经济和应对复杂局面的本领。要进一步解放思想，打破不利于加快发展的思想观念和条条框框，摒弃不利于科学发展的思维定势和行为方式，真正把解放思想的成效体现在克服当前困难、破解发展难题、扫除发展障碍上来。要始终保持奋发有为的精神状态，坚决克服消极畏难、顺势而下的思想情绪，以敢于胜利的坚定信心，攻坚克难的顽强斗志，逆势而上的拼搏精神，推动经济社会又好又快发展。

（二）坚持依法行政，进一步提高科学决策的能力。各级政府及其工作人员要深入贯彻全面推进依法行政实施纲要，认真落实《国务院关于加强市县政府依法行政的决定》，切实增强依法行政、依法办事的意识，自觉运用法律手段解决各种矛盾和问题。进一步落实行政执法责任制和执法过错追究制，建立健全公众参与重大决策的规则和程序，完善行政决策信息和智力支持系统，增强行政决策的透明度和公众参与度。大力推进重大决策听证、公示制度和集体决策制度，促进决策的科学化、民主化、规范化。继续推进相对集中行政处罚权和综合行政执法试点工作，从源头上解决多头执法、重复执法和执法缺位的问题。各级政府要自觉坚持党的领导，主动接受人大依法监督和政协民主监督，认真办理人大代表议案、建议和政协委员提案，广泛听取各民主党派、工商联、无党派人士及各人民团体的建议和意见，鼓励支持人民群众、新闻媒体对政府工作进行监督，促进行政权力依法、公正、有效运行。

（三）坚持务实勤政，进一步提高公共服务的能力。要主动适应体制转轨、社会转型的要求，强化社会管理和公共服务职能，完善公共服务体系，优化公共资源配置，把更多的注意力放到社会管理上，把更多的精力投入到公共服务上，把更多的财力倾斜到改善民生上。深入推进政企分开、政资分开、政事分开，减少政府对微观经济运行的干预，引导和激发各类市场主体的创业活力。各级政府及其工作人员要牢记“两个务必”，大兴求真务实、真抓实干之风，深入实际、深入基层、深入群众，开展调查研究，做到问政于民、问需于民、问计于民。要坚持全心全意为人民服务，满腔热情地解决好人民群众最关心、最直接、最现实的利益问题，真心实意为人民群众做实事、办好事、解难事，努力让人民群众共享改革发展的成果。

（四）坚持高效施政，进一步提高执行落实的能力。切实加强机关效能建设，深化行政审批制度改革，完善行政服务中心功能，扩大“两集中、两到位”试点范围，全面实行重大项目并联审批绿色通道，推行企业全程代理服务。进一步转变工作作风，坚持少开会、开短会，少行文、行短文，少评比、评实效。认真贯彻《政府信息公开条例》，大力推进政务公开，完善新闻发布制度，加快政府网站信息的维护和更新，办好政府公报，及时准确地向社会公开政府信息，确保公民的知情权、参与权、表达权和监督权。加大经济环境治理力度，进一步清理和规范各类行政事业性收费项目，大力整治行政不作为、慢作为、乱作为，严厉打击强装强卸、强买强卖、欺行霸市等扰乱市场秩序的行为。建立工作落实长效机制，健全目标管理考核体系，加强政务督查督办，确保政令畅通。

（五）坚持从严治政，进一步提高拒腐防变的能力。加强政府廉政建设，认真落实党风廉政建设责任制和领导干部廉洁自律的各项规定，完善惩治和预防腐败体系，大力开展政风行风评议，强化行政监察和审计监督，切实纠正部门和行业不正之风，推进土地开发、政府采购、资金安排、国有资产转让、重大工程建设等重点领域、重点环节的制度完善和创新，拓展从源头上防治腐败的工作领域。进一步加大贯彻落实科学发展观、扩内需保增长政策措施以及民生工程实施情况的监督检查，坚决杜绝形象工程和“豆腐渣”工程。牢固树立过紧日子思想，坚持勤俭办一切事业，自觉抵制拜金主义、享乐主义的侵蚀和奢侈浪费现象。各级政府工作人员尤其是领导干部一定要常修为政之德，常思贪欲之害，常怀律己之心，堂堂正正做人，干干净净办事，不断增强拒腐防变能力。各级政府要旗帜鲜明地支持监察部门的工作，对违纪违法案件和腐败分子，坚决严肃查处，决不姑息！

各位代表，新的一年，对于顺利实现“十一五”规划目标至关重要。让我们紧密团结在以胡锦涛同志为总书记的党中央周围，在中共阜阳市委的正确领导下，振奋精神，坚定信心，凝心聚力，开拓进取，不断开创阜阳各项工作新局面，以优异成绩向建国六十周年献礼！
